# Uncemented Customized Hollow Stems in Tumor Endoprosthetic Replacement—A Good Opportunity to Protect the Adjacent Joint in Children?

**DOI:** 10.3390/jpm14090919

**Published:** 2024-08-29

**Authors:** Recep Öztürk, Arne Streitbürger, Jendrik Hardes, Gregor Hauschild, Wiebke K. Guder, Lars Erik Podleska, Markus Nottrott, Nina Myline Engel

**Affiliations:** Department of Orthopedic Oncology, University Hospital Essen, 45147 Essen, Germanygregor.hauschild@uk-essen.de (G.H.); wiebke.guder@uk-essen.de (W.K.G.); ninamyline.engel@uk-essen.de (N.M.E.)

**Keywords:** custom-made endoprosthesis, short stem, hollow stem, 3D printing, individualized medicine, megaendoprosthesis, tumor prosthesis, hydroxyapatite coating

## Abstract

This study aimed to retrospectively analyze the follow-up results of cases in which the adjacent joint was preserved using a custom-made uncemented short-stem design (hollow stem) with optional external flanches in tumor endoprosthetic replacement due to bone sarcomas in 13 patients (with an average age of 9.6 years) between 2017 and 2023. Reconstructions were proximal femur (*n* = 6), intercalary femur (*n* = 4), intercalary tibia (*n* = 2), and proximal humerus (*n* = 1) tumor prostheses. The hollow body was used distally in 10 of the megaprotheses, proximally in 1, and both proximally and distally in 2 of them. The average distance from the joints was 6 cm in stems with flanches and 11.8 cm in stems without flanches. No aseptic loosening or deep infection was observed during an average follow-up of 34 months. Except for one case with a tibial intercalary prosthesis that needed a revision, all cases were well osteointegrated and all lower extremity cases could bear full weight without pain. In cases where the remaining bone stock after bone resection is insufficient for a standard stem implantation, reconstruction with a patient-specific short hollow-stem design appears to be a good alternative to protect healthy joints with high prosthesis survival and low revision rates in the short-term follow-up.

## 1. Introduction

Many methods have been described for osteoarticular and intercalary defect reconstruction after tumor resection in primary malignant bone tumors, such as vascularized fibula autograft, allografts, the removal of the tumor from the resected bone, and reuse by applying radiotherapy or liquid nitrogen, the induced membrane technique, distraction osteogenesis, metallic components, and combinations of these techniques [1,2]. However, due to its advantages such as immediate stability and early weight bearing, reconstruction with megaprostheses is one of the most widely accepted and upcoming options [3]. On the other hand, regardless of the technique applied, there is a consensus in the literature that superior function can be achieved in cases where adjacent joints can be protected. Preserving a joint means leaving the joint-stabilizing ligaments and proprioceptive organs intact, which provides satisfactory functional results [2]. Protecting the epiphysis with customized implants in young patients leads to the prevention of huge leg length discrepancies.

Modular tumor endoprostheses, which are commonly used today, have standardized stems in length and diameter, but these cannot be used in the presence of a short residual bone stock after resection or in small bone anatomy. Shorter stems without special coatings or anchorages, which were developed for such situations, are associated with complications and the joint may have to be sacrificed. This leads to potential increased instability and permanent loss of or reduction in function, and carries the risk of prosthetic complications [4].

However, especially in malignant bone tumors involving a long segment, short-stem designs may be preferred in order not to sacrifice both joints in the presence of short residual bone stock in the proximal and distal parts of the tumor [5]. Advanced porous-coated short-stem designs that can be prepared according to the residual medullary defect can reduce the risks of aseptic loosening and implant failure. Reconstructions with satisfactory function can be achieved [3]. The hollow hexagonal stems developed for these purposes can lead to successful results with protruding fins and the implantation of interlocking screw options. These stems show prospects for improving primary and long-term stability rates compared to standard stems in metaphyseal, metadiaphyseal, and epiphyseal sites [6,7]. This study aimed to retrospectively analyze the follow-up results of megaprosthesis cases in which the adjacent joint was protected using a custom-made porous-coated hollow short-stem design.

## 2. Materials and Methods

In this study, the data of a total of 13 patients who underwent wide tumor resection and reconstruction with modular endoprostheses including hollow stem components were retrospectively analyzed. Inclusion criteria were (1) having a diagnosis of sarcoma; (2) not having sufficient bone stock for a conventional stem and using a custom-made hollow stem; and (3) being under 18 years of age. Patients with (1) less than 12 months of follow-up or (2) missing data were excluded from the study. The patients were treated or followed up between 2010 and 2023 in a tertiary sarcoma center by experienced senior surgeons. Patients’ data were collected anonymously from the patient’s records.

### 2.1. Stem Characteristics

Customized stems were planned using plain radiographs or preoperative computer tomography scans (DICOM format (Implantcast, Buxtehude, Germany), reconstruction matrix 512 × 512, slice thickness ≤1 mm).

The stems were characterized by an adjustable length and diameter depending on remaining bone stock, tapered, non-cemented fixation, titanium–aluminum–vanadium alloy (ISO 5832-3), rough surface with hydroxyapatite coating, hexagonal stem design with protruding fins, and the option of implantation of two interlocking screws [7]. Optional external implant flaps (flanches) increase the contact surface.

### 2.2. Complications

Complications requiring surgical intervention were classified according to the Henderson classification [8]. A height difference of >2 cm between two extremities was considered as extremity inequality.

### 2.3. Implant Survival

Implant survival was defined as the preservation of the prosthesis and adjacent joints, regardless of whether a portion of the implant needed to be replaced in revision surgery. A revision of the entire prosthesis or amputation was defined as reconstruction failure.

### 2.4. Statistical Analysis

All statistical analyses were performed using IBM SPSS 22.0 statistical software (IBM Corp., Armonk, NY, USA). Descriptive statistics are expressed as mean ± standard deviation, frequency, and percentage.

### 2.5. Patient Characteristics

While the overall patient age at the time of the operation was 9.6 years (range 4–15 years), it was 11.8 years (range 9–15 years) in the intercalary prosthesis group and 7.8 years (range 4–11 years) in the other group. All patients (*n* = 13) underwent a biopsy to confirm the diagnosis of a primary bone tumor before the start of oncological treatment. Histological diagnoses were listed as follows: Ewing’s sarcoma (*n* = 7), osteosarcoma (*n* = 3), osteofibrous dysplasia (OFD)-like adamantinoma (*n* = 2), and pleomorphic sarcoma (*n* = 1). Neoadjuvant chemotherapy was applied in 11 patients (all except cases no 7 and no 8). The data of the patients are given in more detail in Table 1.

In three patients, the prosthesis was placed after a prior surgery and after additional (neo-) adjuvant treatment where required. In one of these patients, a cement spacer was applied after proximal humerus resection, and he presented with luxation of the spacer after 62 months (case no 2). A patient with a tumor in the femoral diaphysis underwent a reconstruction with a combination of a fibula allograft and a femoral nail. The patient presented with nonunion and implant failure after 35 months (case no 5). A case of adamantinoma located in the tibia was previously treated with curettage and presented with local recurrence after 47 months (case no 8) (Table 2).

Wide surgical margins were obtained in all patients. Physes were preserved in all cases.

### 2.6. Implant Features

**Figure 1:**
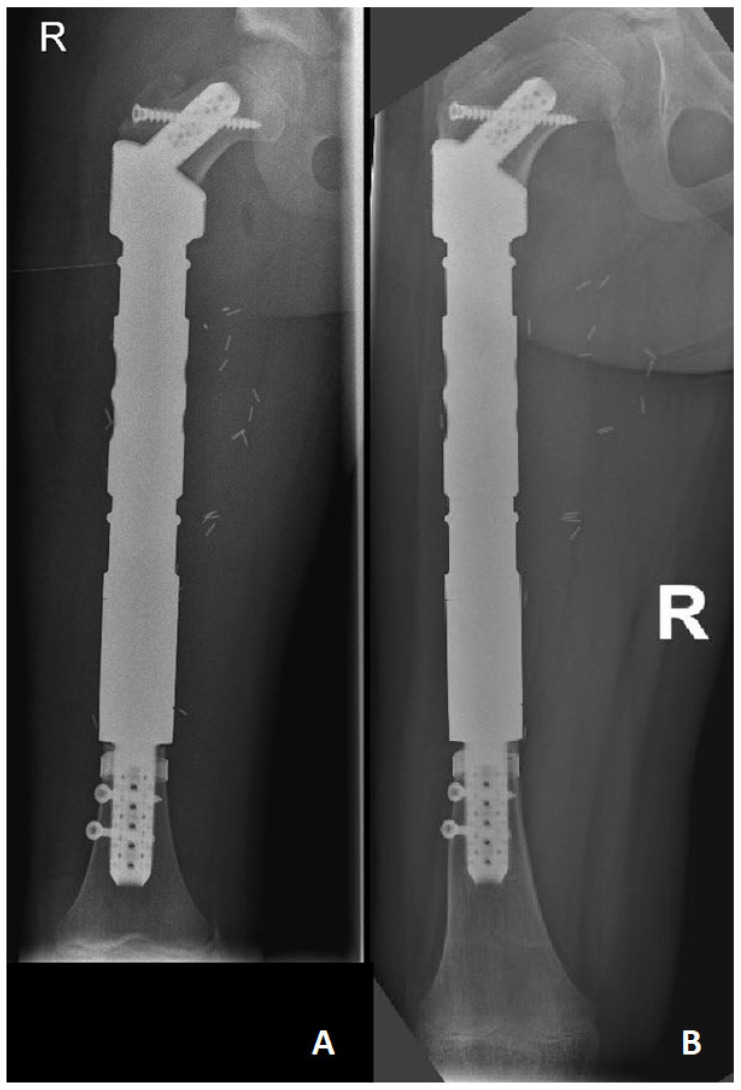
Due to Ewing’s sarcoma located in the right femur diaphysis (case no 6), an intercalary prosthesis was applied using a hollow stem specially prepared for the patient’s defect in the proximal and distal parts. (**A**) Post-operative X-ray of the right femur (a. p.) (**B**) X-ray (a. p.) at 36-month follow-up with no sign of implant failure, stem loosening, or local recurrence.

**Figure 2:**
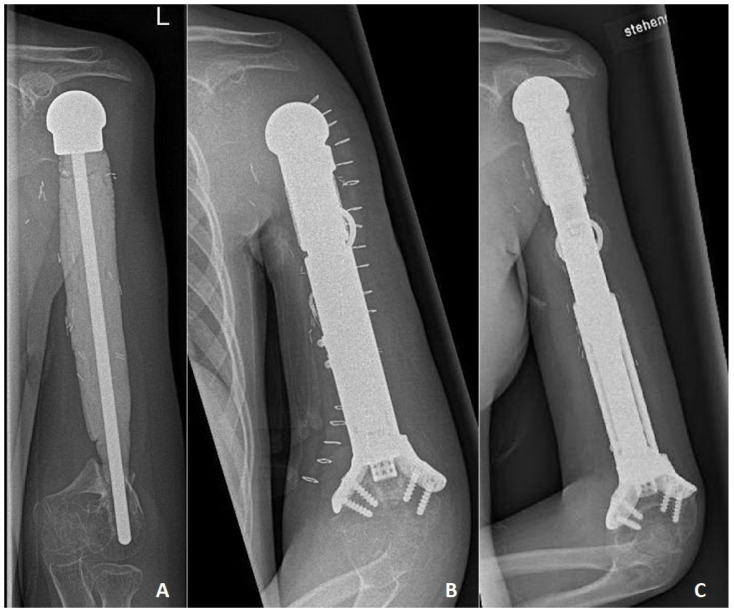
Due to osteosarcoma located in the left proximal humerus (case no 2), a cement spacer was applied after resection, and the patient presented with increased luxation of the distal part of the spacer after 62 months. (**A**) preoperatively a.p. left humerus X-ray. (**B**) X-ray a.p. left humerus, revision and re-construction with a customized expandable prosthesis with distal hollow stem. Locking screws and external implant faps that increase the contact surface and stability. (**C**) X-ray a.p. left humerus at 50-month follow-up (prosthesis was extended by 5 cm).

An intercalary prosthesis was used in six cases (femur: *n* = 4, tibia: *n* = 2), and a joint-containing endoprosthesis was used in seven cases (proximal femur: *n* = 6, proximal humerus: *n* = 1) (Figure 1). All joint-containing endoprostheses mentioned above were combined with a growing option for the future lengthening of the affected limb (Figure 2). While a motor for lengthening was used in the expandable prosthesis in four cases, a placeholder (dummy) was used instead of the motor in three cases, with a 2-stage extension planned (Table 3).

## 3. Results

The average size of the remaining bone after tumor resection was 8 cm (range 3–16.5). The average distance from the joints was 6 cm in stems with flanches and 11.8 cm in stems without flanches. The average resection length and the resulting bone defect was 25.8 cm (range 17–32 cm). The average surgery duration was 205 min (range 116–325 min).

At a mean follow-up of 33.9 months (range 6–65 months) after surgery, eleven patients are currently alive with no evidence of disease (NED). One patient died due to the progression of the disease (case no 13) and one due to chemotherapy toxicity (case no 10), at 38 and 6 months, respectively. The average follow-up period of the surviving patients is 35.9 months (range 12–65 months). No patient developed local recurrence during follow-up.

### 3.1. Hollow-Stem-Related Complications

Only one hollow-stem-related complication was encountered. Tibial bowing, tilt, and irregular gait along with partial consolidation (Henderson type 2) were observed in one patient (case no 7) who underwent intercalary tibia prosthesis. A revision is planned when growth is completed.

### 3.2. Other Complications

A total of eight complications (intercalary prosthesis: *n* = 2, prosthesis with joint reconstruction: *n* = 6) occurred in this case series.

Soft tissue failure was observed in two patients (Henderson type I). Wound healing disorders without the involvement of the implant were treated successfully by wound revision.

A patient with a proximal femoral prosthesis and a distal hollow stem developed chronic hip subluxation which was treated through the implantation of a cup and the tenotomy of the proximal adductors at the 31st month (case no 1). A temporary hemiepiphysiodesis of the medial distal femur was necessary in a patient with a proximal femoral prosthesis who developed ipsilateral genu valgum 22 months postoperatively (case no 4).

Four complications did not require surgery and were followed up: joint subluxation in two patients (proximal femoral prosthesis: *n* = 1, proximal humerus prosthesis: *n* = 1) and genu valgum in two patients.

No periprosthetic infection (Henderson type 4) or local recurrence (Henderson type 5) was observed in this small cohort. Limb salvage was achieved in all patients. Except for the case with a tibial intercalary prosthesis that had partial consolidation and for which a revision is planned, all cases had intact implants and all lower extremity cases could bear full weight without pain. In conclusion, only one patient presented with a hollow-stem-specific complication. As mentioned above, two patients died with an intact implant in an average of 22 months. With an average follow-up of 33.9 months, the average reconstruction survival was 92% (*n* = 12). Complications are given in Table 1.

Proximal and distal epiphyses were preserved in all patients who underwent intercalary prostheses, and no inequality developed in any of them. Three patients with expandable prostheses were extended a total of 8.5 cm (5 cm, 1 cm, and 2.5 cm), and none of them had limb discrepancy at the last examination. In one patient where a proximal femur endoprosthesis was applied (case no 4), there was a shortness of 2.8 cm at the last check-up and his follow-up continues. Hip subluxation developed in a 4-year-old child (case no 12) with Fanconi syndrome and bilateral genu valgum. The expandable prosthesis could not be lengthened due to cable failure in this case, and at the last check-up, there was approximately 5 cm of limb shortening. A revision was planned for the patient around the age of 12.

## 4. Discussion

Recently, with the developments in imaging techniques, neoadjuvant chemo-radiotherapies, and surgical techniques, limb-sparing treatment has become the standard treatment for malignant bone and soft tissue sarcomas. Endoprostheses have important advantages over biological methods, such as immediate stability, early weight bearing, and rapid rehabilitation. However, standardized stems cannot be used in the presence of short residual bone stock due to massive defects [3]. Short stems with the same design as a conventional stem were initially associated with aseptic loosening [9,10]. However, Stevenson et al. reported the possibility of adding an extracortical plate to the developed cemented short-stem design with similar results compared to standard-size stems. On the other hand, in this design, extracortical plate addition is required in all cases, and more bone had to be exposed to place this plate; this method also means more implant load and increased soft tissue detachment to place the plate [9]. In two studies reporting the results of another short-stem design, revision surgeries were reported at 30% and 50%, respectively [11,12]. Cannon et al. published 9% failure rates with their custom-made cemented cross-pin stem design, where the majority of the cases were short stems [13]. Due to the described high failure rates, the long-term survival of these designs was viewed critically [3]. With the recently developed three-dimensional-printed (3DP) prosthesis designs, stems can be produced according to the medullary cavity of the patient’s residual bone. The ability to produce customized implants that mimic anatomy in complex anatomical and biomechanical regions increases joint and limb salvage rates in both osteoarticular and intercalary reconstructions [1,3,14,15]. In 2023, Li et al. reported aseptic loosening in 1 of 12 cases (9%) in which they used porous-coated uncemented prosthesis designs [4]. In the current study, we also used a cementless design, a 3D HA-coated prosthesis with a porous, hollow stem. The most important result of this study is that high prosthetic and extremity survival was achieved along with low complication rates. It is important to note that these results were obtained in a patient group where one part of the cases involved expandable prostheses of growing children and the other part involved intercalary prostheses.

In a hollow-stem design, in the early postoperative period, the stem movement is limited by the bone inside and outside. In addition, locking screws and external implant faps can increase the contact surface. In this way, stability can be increased. Especially in an area with a large surface such as the metaphysis and where the cancellous bone is located, a hard and solid stem can crush the cancellous tissue, move within the cancellous bone, and cause an angulation between the bone and the prosthesis, or a fracture may occur. However, a hollow-stem design will be more stable thanks to the bone tissue inside and around the stem. In addition, the special inner and outer surfaces create more bone–prosthesis integration surface area. In the later postoperative period, the presence of more bone stock integrated into the prosthesis, including the medullary space, may create a more stable structure. The results of the current study show that this design is feasible.

It is known that the risk of complications is higher in intercalary prostheses compared to endoprostheses containing joint components, due to the presence of different mechanisms such as shear forces [7]. Except for the partial consolidation and angulation seen in only one patient, the fact that full osteointegration developed in all other patients shows that the hollow-stem design with a specially coated surface can be used for intercalary reconstructions. In addition, in our study, the problem of limb inequality was not seen in any case in the intercalary prosthesis group, where both adjacent physes/joints could be protected. Incidentally, all patients in this study were children, and the complete survival of the prosthesis was observed in all cases where a noninvasive expandable prosthesis was used. As is known, noninvasive expandable prostheses have their own complications. In a meta-analysis, Portney et al. showed that mechanical complications were especially higher in noninvasive prostheses than in minimal invasive prostheses [16]. Limb inequality is a common problem in young patients with healthy epiphysis. This problem is aggravated by using standardized prostheses. Hollow stems make a serious contribution to the solution of this problem by protecting at least one healthy physis/joint. While no lengthening was required at the last follow-up in two of the seven cases in which an expandable prosthesis was used, the extension of the prosthesis was performed in three cases and the limb inequality was eliminated. The follow-up and treatment process actually continues in two patients with limb inequality. These results show that reconstruction with a hollow-stem design can be successfully combined with expandable prostheses with acceptable complication rates. Hollow stems reduce the need for limb lengthening procedures if a joint and an epiphysis can be preserved with this stem design. As a result, the fact that hollow stems can be used with an expandable prosthesis component and extensions can be made without any additional surgery shows that these stems are a good alternative for protecting the physis and joints.

Femoral diaphyseal tumors close to both joints were an indication of total femoral replacements in the past in our department. This leads to the deterioration of all the muscles in the proximal femur, leading to poor functional outcomes at the hip, including Trendelenburg claudication. A risk of luxation of the hip is also present. In addition, total femur prosthesis also includes problems arising from distal femur resection. In these cases, complications such as periprosthetic infection, aseptic loosening, periprosthetic fracture, and material wear may occur [4,6]. On the other hand, total tibial prosthesis has not become widespread in cases of tibial diaphyseal tumors close to both joints. All this information suggests that using a hollow stem is a good option to prevent or postpone joint resection and it reduces secondary implant-related complications because joint replacement is not necessary.

This study had some limitations. First of all, the study had a retrospective design and did not have functional scoring data. The case series results of an advanced implant design from a specialized and well-equipped tumor center contribute to the literature, but clear comparisons could not be made due to the individual character of the implants and the heterogeneous patient cohort. Additionally, the relatively small number of cases including the results of a single center limits the power of the data. Moreover, although all patients, except for one patient who died at 6 months, were followed for at least 1 year, the average follow-up period was relatively short. Future multicenter comparative studies with larger patient numbers and longer follow-up periods will help us better understand the results of modern short-stem designs and the management of complications.

## 5. Conclusions

In cases where the remaining bone stock after bone resection is insufficient for the implantation of a conventional stem, reconstruction with a customized short hollow-stem design appears to be a good alternative with high prosthesis survival and low revision rates in the short-term follow-up. It is promising that these results were obtained especially in the patient group with open epiphysis and the intercalary prosthesis group. The preservation of the adjacent joint and the epiphysis with a hollow stem design improves functional outcomes. Preserving epiphyses leads to less limb length discrepancy after joint replacement.

## Figures and Tables

**Table 1:** Case characteristics, management, and outcome.

Case No	Age	Pr/R	Localization	Diagnosis	Reconstruction with TP	FU for TP *	Complication	Last Status
1	8	Pr	Femur proximal	Ewing’s sarcoma	Xpandable proximal femur	47	Chronic hip subluxation	NED
2	10	R	Humerus proximal	Osteosarcoma	Xpandable proximal humerus	50	Chronic shoulder subluxation	NED
3	10	Pr	Femur proximal	Osteosarcoma	Xpandable proximal femur	42	genu valgum	NED
4	7	Pr	Femur proximal	Ewing’s sarcoma	Xpandable proximal femur	40	genu valgum	NED
5	15	R	Femur diaphysis	Osteosarcoma	Intercalary femur	29	-	NED
6	9	Pr	Femur diaphysis	Ewing’s sarcoma	Intercalary femur	36	-	NED
7	9	Pr	Tibia diaphysis	OFD-like adamantinoma	Intercalary tibia	29	Tibial bowing, tilt, wound healing disorder	NED
8	15	R	Tibia diaphysis	OFD-like adamantinoma	Intercalary tibia	25	-	NED
9	12	Pr	Femur diaphysis	Ewing’s sarcoma	Intercalary femur	20	Wound healing disorder	NED
10	11	Pr	Femur diaphysis	Pleomorphic sarcoma	Intercalary femur	6		DOD
11	11	Pr	Femur proximal	Ewing’s sarcoma	Xpandable proximal femur	12	-	NED
12	4	Pr	Femur proximal	Ewing’s sarcoma	Xpandable proximal femur	65	Hip subluxation, genu valgum	NED
13	4	Pr	Femur proximal	Ewing’s sarcoma	Xpandable proximal femur	40		DOD

Pr: primary surgery; R: revision surgery; TP: tumor prosthesis; NED: no evidence of disease; DOD: died of disease; * months.

**Table 2:** Previous surgeries and revisions with prostheses included hollow stems.

Case No	Previous Operation	Indication	Prosthesis Operation
2	Cement-spacer implantation	Increased dislocation at the distal part of the spacer	Custom-made proximal humerus tumor prosthesis
5	Fibula allograft, intramedullary nail	Nonunion, implant failure	Implant removal + intercalary femur prosthesis
8	Curettage, plate osteosynthesis	Local recurrence	Wide resection + intercalary tibia prosthesis

**Table 3:** Hollow stem, expandable prosthesis components, extension of the prosthesis (if applicable), and leg length discrepancy.

Case No	Hollow Stem Localization	Motor or Placeholder	Extension (cm)	Complication of Extension	Limb Inequality
1	distal	motor	1	-	1.5
2	distal	motor	5	-	-
3	distal	placeholder	-	-	2.0
4	distal	placeholder	-	-	2.8
5	distal	-	-	-	-
6	proximal and distal	-	-	-	-
7	proximal	-	-	-	-
8	proximal and distal	-	-	-	-
9	distal	-	-	-	-
10	distal	-	-	-	-
11	distal	placeholder	-	-	-
12	distal	motor	-	cable failure	5
13	distal	motor	2.5	-	0.5

## Data Availability

The data presented in this study are available on reasonable request from the corresponding author.

## References

[1] Wang J., An J., Lu M., Zhang Y., Lin J., Luo Y., Zhou Y., Min L., Tu C. (2021). Is three-dimensional-printed custom-made ultra-short stem with a porous structure an acceptable reconstructive alternative in peri-knee metaphysis for the tumorous bone defect?. World J. Surg. Oncol..

[2] Fuchs B., Ossendorf C., Leerapun T., Sim F.H. (2008). Intercalary segmental reconstruction after bone tumor resection. Eur. J. Surg. Oncol..

[3] Liu W., Shao Z., Rai S., Hu B., Wu Q., Hu H., Zhang S., Wang B. (2020). Three-dimensional-printed intercalary prosthesis for the reconstruction of large bone defect after joint-preserving tumor resection. J. Surg. Oncol..

[4] Li Z., Lu M., Zhang Y., Gong T., Min L., Zhou Y., Luo Y., Tu C. (2023). 3D-printed custom-made short stem with porous structure for fixation of massive endoprosthesis in joint-preserving reconstruction after tumor resection. J. Orthop. Surg. Res..

[5] Qu H., Guo W., Yang R., Tang X., Yan T., Li D., Yang Y., Zang J. (2015). Cortical strut bone grafting and long-stem endoprosthetic reconstruction following massive bone tumour resection in the lower limb. Bone Jt. J..

[6] Dieckmann R., Henrichs M.P., Gosheger G., Höll S., Hardes J., Streitbürger A. (2014). Short-stem reconstruction for megaendoprostheses in case of an ultrashort proximal femur. BMC Musculoskelet. Disord..

[7] Streitbürger A., Hardes J., Nottrott M., Guder W.K. (2022). Reconstruction survival of segmental megaendoprostheses: A retrospective analysis of 28 patients treated for intercalary bone defects after musculoskeletal tumor resections. Arch. Orthop. Trauma Surg..

[8] Pala E., Henderson E.R., Calabrò T., Angelini A., Abati C.N., Trovarelli G., Ruggieri P. (2013). Survival of current production tumor endoprostheses: Complications, functional results, and a comparative statistical analysis. J. Surg. Oncol..

[9] Stevenson J.D., Wigley C., Burton H., Ghezelayagh S., Morris G., Evans S., Parry M., Jeys L. (2017). Minimising aseptic loosening in extreme bone resections: Custom-made tumour endoprostheses with short medullary stems and extra-cortical plates. Bone Jt. J..

[10] Sewell M.D., Hanna S.A., McGrath A., Aston W.J., Blunn G.W., Pollock R.C., Skinner J.A., Cannon S.R., Briggs T.W. (2011). Intercalary diaphyseal endoprosthetic reconstruction for malignant tibial bone tumours. J. Bone Jt. Surg. Br..

[11] Calvert G.T., Cummings J.E., Bowles A.J., Jones K.B., Wurtz L.D., Randall R.L. (2014). A dual-center review of compressive osseointegration for fixation of massive endoprosthetics: 2- to 9-year followup. Clin. Orthop. Relat. Res..

[12] Goulding K.A., Schwartz A., Hattrup S.J., Randall R.L., Lee D., Rispoli D.M., Lerman D.M., Beauchamp C. (2017). Use of compressive osseointegration endoprostheses for massive bone loss from tumor and failed arthroplasty: A viable option in the upper extremity. Clin. Orthop. Relat. Res..

[13] Cannon C.P., Eckardt J.J., Kabo J.M., Ward W.G., Kelly C.M., Wirganowicz P.Z., Asavamongkolkul A., Nieves R., Eilber F.R. (2003). Custom cross-pin fixation of 32 tumor endoprostheses stems. Clin. Orthop. Relat. Res..

[14] Zhao D., Tang F., Min L., Lu M., Wang J., Zhang Y., Zhao K., Zhou Y., Luo Y., Tu C. (2020). Intercalary reconstruction of the “ultra-critical sized bone defect” by 3D-printed porous prosthesis after resection of tibial malignant tumor. Cancer Manag. Res..

[15] Shao X., Dou M., Yang Q., Li J., Zhang A., Yao Y., Chu Q., Li K., Li Z. (2023). Reconstruction of massive bone defects after femoral tumor resection using two new-designed 3D-printed intercalary prostheses: A clinical analytic studywith the cooperative utilization of multiple technologies. BMC Musculoskelet. Disord..

[16] Portney D.A., Bi A.S., Christian R.A., Butler B.A., Peabody T.D. (2020). Outcomes of Expandable Prostheses for Primary Bone Malignancies in Skeletally Immature Patients: A Systematic Review and Pooled Data Analysis. J. Pediatr. Orthop..

